# Sex-specific prevalence and risk factors of metabolic-associated fatty liver disease among 75,570 individuals in eastern China

**DOI:** 10.3389/fendo.2023.1241169

**Published:** 2023-09-26

**Authors:** Mingxing Chang, Zhihao Shao, Wei Wei, Peipu Shen, Guifang Shen

**Affiliations:** Department of Health Management Center, The Affiliated Hospital of Xuzhou Medical University, Xuzhou, Jiangsu, China

**Keywords:** MAFLD, sex-specific, metabolic disorder, prevalence, risk factor

## Abstract

**Background:**

Metabolic-associated fatty liver disease (MAFLD) is a newly proposed definition and there is limited data on MAFLD prevalence. We aimed to investigate the prevalence of MAFLD in an eastern Chinese population.

**Methods:**

This cross-sectional study included participants from an eastern Chinese population who underwent regular health checkups. Based on current diagnostic criteria, MAFLD was diagnosed in individuals with both hepatic steatosis and metabolic disorders. The overall and stratified prevalence derived based on sex, age, body mass index (BMI), and various metabolic disorders were estimated. Multivariate logistic regression analysis was used to determine the risk factors for MAFLD.

**Results:**

Among the 75,570 participants, the overall prevalence of MAFLD was 37.32%, with higher rates in men (45.66%) than in women (23.91%). MAFLD prevalence was highest in men aged 40–49 years (52.21%) and women aged 70–79 years (44.77%). In all the BMI subgroups, the prevalence was higher in men than in women. In both sexes, the prevalence of MAFLD increased as BMI levels increased. Furthermore, MAFLD was associated with metabolic disorders, especially in the female participants with severe obesity (odds ratio 58.318; 95% confidence interval: 46.978–72.397).

**Conclusion:**

MAFLD is prevalent in the general adult population in eastern China. Sex-specific differences in MAFLD prevalence were identified based on age, BMI, and metabolic disorders. MAFLD is associated with metabolic disorders, particularly obesity.

## 1 Introduction

Metabolic-associated fatty liver disease (MAFLD), previously known as nonalcoholic fatty liver disease (NAFLD), has recently gained substantial attention because of its high prevalence and serious health consequences (1, 2). Although NAFLD was initially considered a liver-specific disease, research has shown the association of NAFLD with various metabolic dysfunctions and complex pathophysiological characteristics that potentially lead to liver or cardiovascular outcomes (3). Recently, an international expert panel recommended a novel terminology, MAFLD, to reflect the metabolic association and potentially increase awareness and reduce stigma (4). NAFLD encompasses a broad spectrum of disease severity, ranging from mild steatosis to advanced fibrosis and cirrhosis, whereas MAFLD can better identify individuals with a higher risk of metabolic and cardiovascular morbidity and mortality (5).

The burden of MAFLD is particularly heavy in China, and it is estimated that 315 million people would be affected by MAFLD by 2030. MAFLD is the most common cause of chronic liver disease (6). However, data on the prevalence and risk factors of MAFLD in Xuzhou, China, over the past decade are lacking. Previous studies conducted in Central and Southwest China were limited by small sample sizes, non-resident populations, and incomplete representation (7–9). Moreover, owing to uneven economic development and diverse lifestyles among provinces in China, regional differences in the epidemiology of NAFLD are notable. Therefore, large-scale epidemiological investigations are needed to provide a comprehensive understanding of the disease burden of MAFLD in Xuzhou and to identify high-risk populations.

This cross-sectional comparative study aimed to investigate the prevalence of MAFLD based on the novel diagnostic criteria. We aimed to provide a more comprehensive understanding of the association between MAFLD and multiple metabolic disorders to help contribute to improved management and prevention of MAFLD.

## 2 Materials and methods

### 2.1 Study design and participants

**Figure 1:**
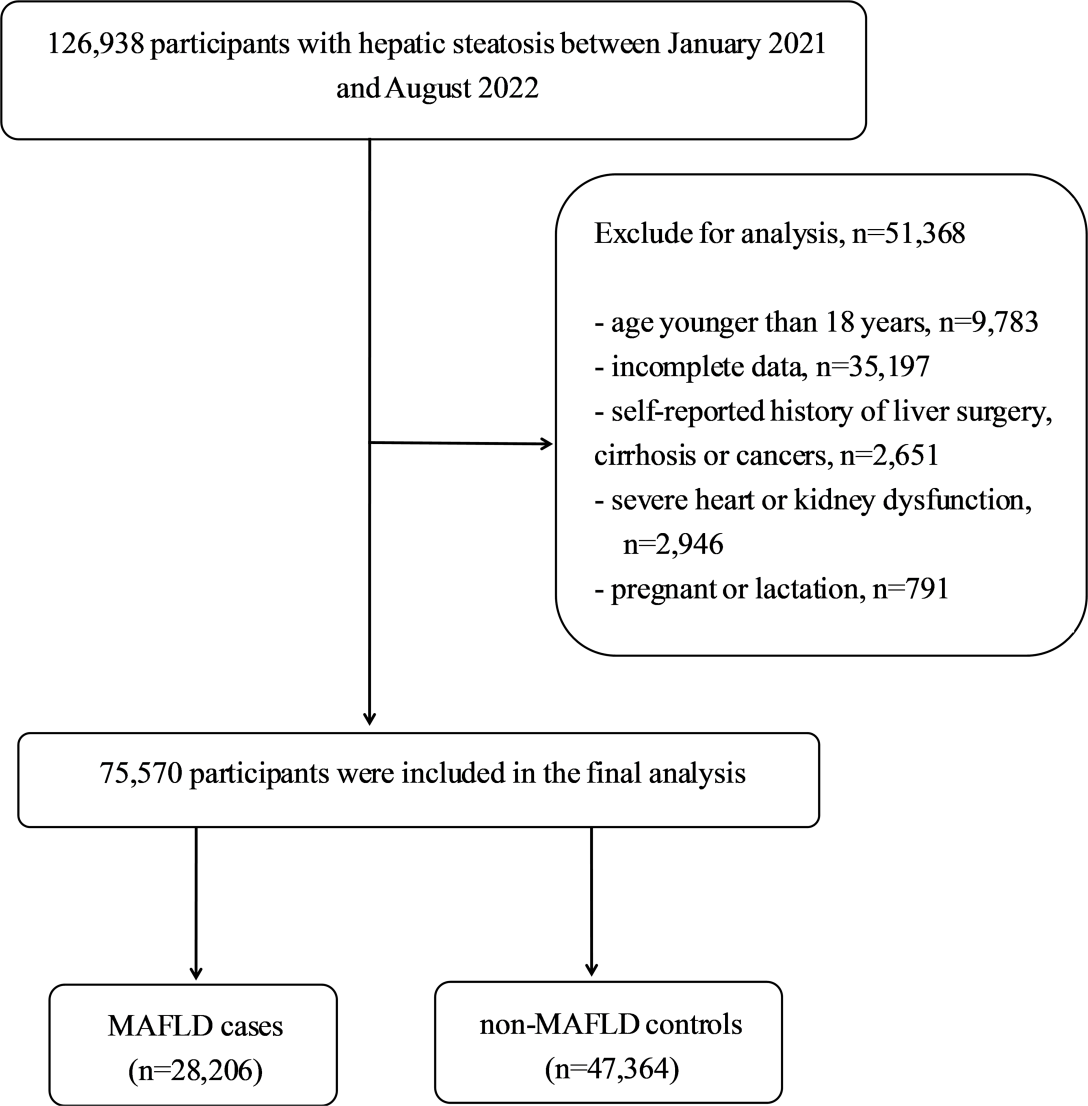
Flowchart of the study design.

This cross-sectional study used data from an urban population in eastern China who underwent regular health checkups at the Affiliated Hospital of Xuzhou Medical University between January 2021 and August 2022. To be included in the study, participants had to be older than 18 years, should have lived in Xuzhou for at least 5 years, and had to have a diagnosis of hepatic steatosis, confirmed through abdominal ultrasound. The exclusion criteria included lack of complete data, cirrhosis, hepatocellular carcinoma, or a history of liver surgery or malignancy; New York Heart Association Class III or IV heart failure; chronic kidney disease with an estimated glomerular filtration rate less than 60mL/min/1.73m^2^; pregnancy or lactation; and missing outcome measures or lost clinical and biochemical records. **Figure 1** shows the flowchart of the participant selection process in the study. To prevent data duplication, only the initial physical examination data of the participants who underwent multiple physical examinations throughout the year were used. Ethical approval was obtained from the Ethics Committee of the Affiliated Hospital of Xuzhou Medical University (approval number: XYFY2023-KL086-01), and the requirement for informed consent was waived because of deidentified data were analyzed in this retrospective study.

### 2.2 Anthropometric measurements and clinical examination

This study followed a standard measurement method, which included a physical examination conducted by a professional doctor to measure height, weight, and blood pressure (BP). The body mass index (BMI) was calculated using the following formula: BMI (kg/m²) = weight (kg)/height² (m²). Laboratory data, including fasting plasma glucose (FPG), triglyceride (TG), total cholesterol (TC), high-density lipoprotein cholesterol (HDL-C), low-density lipoprotein cholesterol (LDL-C), alanine aminotransferase (ALT), aspartate transaminase (AST),-glutamyltransferase (GGT), blood urea nitrogen (BUN), creatinine (Cr), and uric acid (UA) levels, were collected after at least an 8-hour fast during the health examinations. Abdominal ultrasound was performed using ultrasound scanners (Affiniti70, Philips Medical Systems, USA), and steatosis was diagnosed based on ultrasonographic patterns such as liver parenchymal brightness, increased echo contrast between the hepatic and renal parenchyma, and vascular blurring or poor visualization of the diaphragm (10). Moreover, disease history was checked from each participant’s health examination results.

### 2.3 Diagnosis of MAFLD

In this study, we used a novel and positive set of criteria to diagnose MAFLD regardless of coexisting liver disease or alcohol consumption (4). The diagnosis of MAFLD was primarily based on the ultrasonographic detection of hepatic steatosis, in addition to at least one of the following three criteria: overweight or obese, clinical evidence of metabolic dysfunction, or type 2 diabetes mellitus (T2DM). Metabolic dysfunction was determined by the presence of at least two of the following metabolic risk factors: (1) waist circumference ≥90/80 cm for Asian men and women, respectively; (2) BP ≥130/85 mmHg or the specific use of anti-hypertensive medication; (3) TG≥1.70 mmol/L or the specific use of medications for hypertriglyceridemia; (4) HDL-C <1.0 mmol/L for men and <1.3 mmol/L for women or the specific use of medications to elevate HDL-C; (5) prediabetes (i.e., FPG 5.6–6.9 mmol/L, or 2-h post-load glucose levels of 7.8–11.0 mmol/L, or a glycated hemoglobin (HbA1c) of 5.7%–6.4%); (6) high-sensitivity C-reactive protein levels >2 mg/L; and (7) homeostasis model assessment-insulin resistance scores ≥2.5.

### 2.4 Definitions

The BMI categories, including underweight (<18.5 kg/m²), normal (≥18.5 to <23 kg/m²), overweight (≥23 to <25 kg/m²), obese I (≥25 to <29.9 kg/m²), and obese Π (≥30 kg/m²), were determined using the BMI criteria for Asians established by the regional office for the Western Pacific Region of the WHO (11). Hypertension (HTN) was diagnosed based on a self-reported history of HTN determined previously by a healthcare professional if systolic blood pressure (SBP) was ≥140 mmHg and/or diastolic blood pressure (DBP) was ≥90 mmHg, or if a patient was taking specific drug treatment (12). T2DM was defined as having a self-reported history of diabetes determined by a healthcare professional or FPG levels ≥7.0 mmol/L, or if a patient was taking specific drug treatments (13). Dyslipidemia was defined according to the guidelines for the prevention and treatment of dyslipidemia in Chinese adults, with the following criteria: (1) TC ≥5.2 mmol/L; (2) LDL-C ≥3.4 mmol/L; (3) HDL-C <1.0 mmol/L; (4) TG ≥1.7 mmol/L (14).

### 2.5 Statistical analysis

Statistical analyses were conducted using SPSS version 22.0 (IBM Corp., Armonk, NY, USA). Descriptive statistics were used to present continuous variables as mean ± standard deviation (SD) or medians with interquartile ranges (IQR) and categorical variables as proportions (%). The Student’s *t*-test or Mann–Whitney U test was used to compare differences between individuals with and without MAFLD for continuous variables, whereas the chi-square test was used for categorical variables. Prevalence rates and 95% confidence intervals (CI) were calculated and analyzed for the different age, BMI, and metabolic-disorder groups. Binary logistic regression analysis was employed to explore the related risk factors for MAFLD and to calculate the corresponding odds ratios (OR) and 95% CI.

## 3 Results

### 3.1 Clinical and biochemical characteristics of the participants

A total of 75,570 participants were analyzed, comprising 46,610 males (61.68%) and 28,960 females (38.32%). The baseline characteristics of the study participants are presented in **Table 1**. The median ages of those with and without MAFLD were 46 years (IQR:36–56 years) and 41 years (IQR:32–54 years), respectively (p<0.0001). Among all participants, 28,206 (37.32%) were diagnosed with MAFLD and 47,364 served as non-MAFLD controls. The prevalence of MAFLD was significantly higher in males (n=21,281, 45.66%) than in females (n=6,925, 23.91%) (P<0.0001). In males and females, significant differences in blood pressure, BMI, and levels of FPG, TC, TG, LDL-C, ALT, AST, GGT, BUN, Cr, and UA (all P<0.0001) were observed between those with and without MAFLD. The proportion of metabolic disorders, including obesity, T2DM, HTN, and dyslipidemia, was significantly higher in the MAFLD group than in the non-MAFLD group (all *p*<0.0001). Among participants with MAFLD, the prevalence of obesity was 80.53% and 65.34% in males and females, respectively, and this indicated that obesity was the most prevalent metabolic condition.

**Table 1:** Baseline characteristics of the MAFLD and non-MAFLD groups.

Variables	Female		P-value	Male		P-value
	Non-MAFLD	MAFLD		Non-MAFLD	MAFLD	
	(n=22,035)	(n=6,925)		(n=25,329)	(n=21,281)	
General information						
Age (years) BMI (kg/m2) SBP (mmHg) DBP (mmHg)	40 (32,52) 21.99 ± 2.55 118.44 ± 16.58 71.63 ± 10.02	53 (41,60) 26.34 ± 2.92 132.47 ± 18.72 78.15 ± 10.97	<0.0001 <0.0001 <0.0001 <0.0001	42 (32,55) 24.03 ± 2.88 127.91 ± 16.46 78.16 ± 10.91	44 (35,54) 27.41 ± 2.93 133.14 ± 15.86 83.11 ± 11.12	<0.0001 <0.0001 <0.0001 <0.0001
Glucolipid metabolism						
FPG (mmol/L) TC (mmol/L) TG (mmol/L) HDL (mmol/L) LDL (mmol/L)	4.97 (4.68,5.28) 4.57 ± 0.95 0.92 (0.7,1.26) 1.49 (1.3,1.71) 2.85 ± 0.71	5.31 (4.94,5.83) 4.94 ± 1.01 1.6 (1.15,2.22) 1.31 (1.15,1.5) 3.2 ± 0.74	<0.0001 <0.0001 <0.0001 <0.0001 <0.0001	5.14 (4.82,5.53) 4.51 ± 0.91 1.22 (0.89,1.72) 1.25 (1.1,1.43) 2.91 ± 0.69	5.36 (4.98,5.88) 4.78 ± 0.97 1.87 (1.32,2.72) 1.14 (1.01,1.29) 3.12 ± 0.7	<0.0001 <0.0001 <0.0001 <0.0001 <0.0001
Liver function						
ALT (U/L) AST (U/L) GGT (U/L)	13 (10,17) 17 (15,21) 14 (11,18)	18 (14,25) 19 (16,23) 20 (15,28)	<0.0001 <0.0001 <0.0001	19 (15,27) 19 (17,23) 23 (18,34)	27 (20,40) 22 (18,27) 35 (25,54)	<0.0001 <0.0001 <0.0001
Kidney function						
BUN (mmol/L) Cr (µmo/L) UA (µmo/L)	4.52 ± 1.18 54.43 ± 8.27 255.55 ± 55.55	4.79 ± 1.25 54.02 ± 8.94 294.33 ± 65.53	<0.0001 0.001 <0.0001	5.27 ± 1.29 73.35 ± 14.38 348.13 ± 73.88	5.27 ± 1.25 72.83 ± 11.56 382.98 ± 81.65	0.521 <0.0001 <0.0001
Metabolic disorder						
Obesity n (%) T2DM n (%) HTN n (%) Dyslipidemia n (%)	2,611 (11.85%) 362 (1.64%) 2,938 (13.33%) 6,949 (31.54%)	4,525 (65.34%) 659 (9.52%) 2,692 (38.87%) 4,565 (65.92%)	<0.0001 <0.0001 <0.0001 <0.0001	8,772 (34.63%) 1,501 (5.93%) 7,172 (28.32%) 11,654 (46.01%)	17,137 (80.53%) 2,325 (10.93%) 9,206 (43.26%) 15,748 (74%)	<0.0001 <0.0001 <0.0001 <0.0001

Data are expressed as mean±SD or medians (IQRs) for skewed variables or numbers (proportions) for categorical variables. ALT, alanine aminotransferase; AST, aspartate transaminase; BMI, body mass index; BUN, blood urea nitrogen; Cr, creatinine; DBP, diastolic blood pressure; FPG, fasting plasma glucose; GGT, γ‐glutamyltransferase; HDL-C, high-density lipoprotein cholesterol; HTN, hypertension; LDL-C, low-density lipoprotein cholesterol; MAFLD, metabolic-associated fatty liver disease; SBP, systolic blood pressure; TC, total cholesterol; T2DM, type 2 diabetes mellitus; TG, triglyceride; UA, uric acid.

### 3.2 Prevalence of MAFLD and stratification by sex, age, BMI, and metabolic disorder

**Figure 2:**
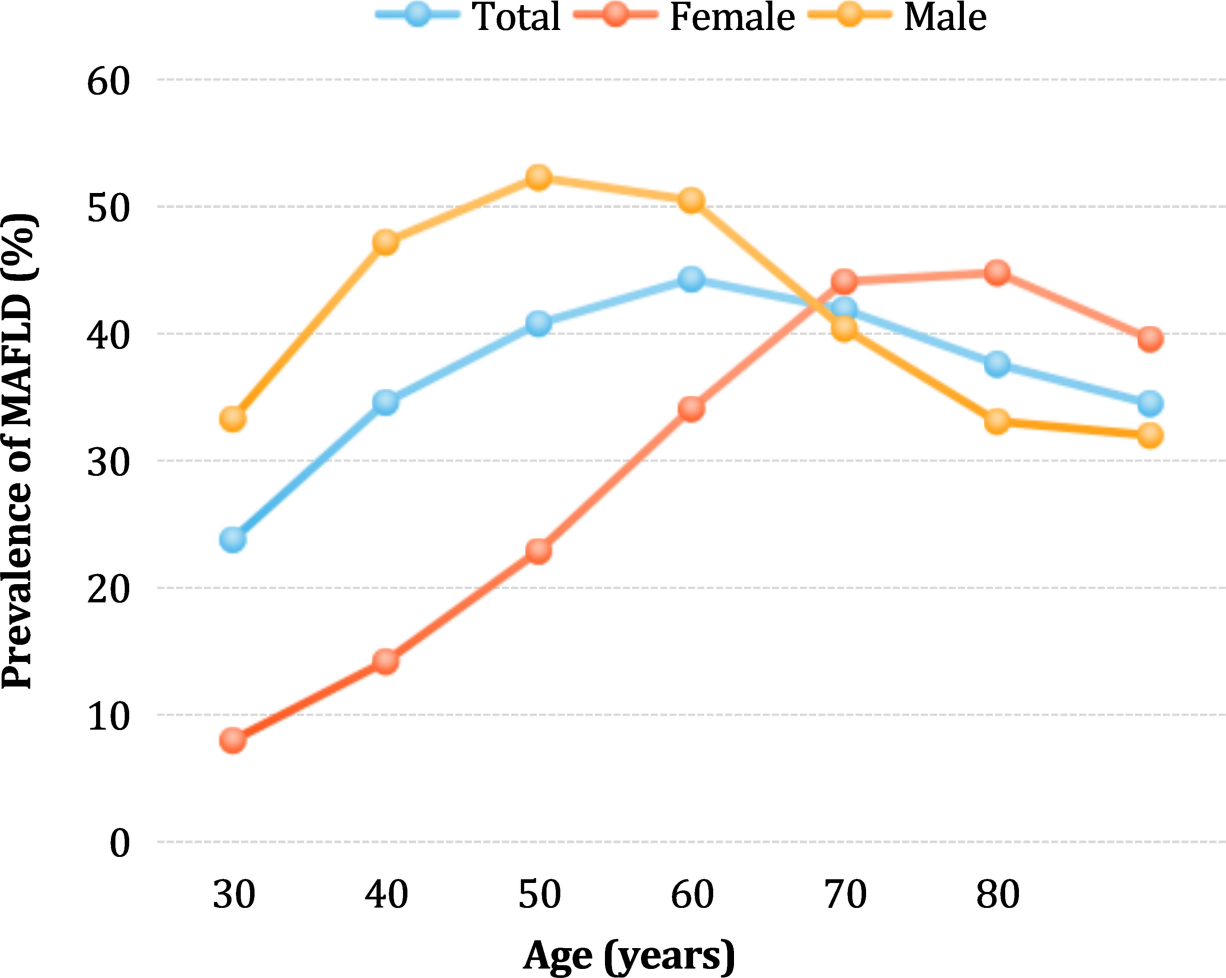
Sex-stratified prevalence of MAFLD in different age groups.

The MAFLD prevalence was not linearly associated with age (**Figure 2**). In the overall population, the prevalence gradually increased with age, peaking at 44.27% in the age group 50–59 years, and then slightly decreased. Interestingly, in the male group, the prevalence rate rapidly increased between the ages of 18–39, then slowed down, with a peak rate of 52.21% in the age group of 40–49 years. After 50 years of age, the prevalence rate gradually decreased, and the lowest rate of 31.91% was found in the group of participants aged over 80 years. In contrast, in females, the lowest prevalence was 8% among those under 30 years of age. The prevalence rate of MAFLD in the female group gradually increased in the 18–49 years age range and rapidly increased after the age of 50 years, reaching a peak of 44.77% between the ages of 70-79. Before the age of 65, the prevalence rate was significantly higher in males than in females (46.67% vs. 21.77%, χ2 value=68603, *p*<0.0001), whereas after age 65, the prevalence rate was significantly lower in males than in females (35.71% vs. 45.06%, χ2 value=6967, *p*<0.0001).

When stratified by BMI and metabolic disorders, sex-specific differences were observed in the prevalence of MAFLD. In the total population, the prevalence rates of MAFLD among individuals with underweight/normal, overweight, and obesity were 4%, 32%, and 65.5%, respectively. Interestingly, men had a considerably higher prevalence of MAFLD than women across all BMI categories (all *p*<0.0001). The prevalence of MAFLD increased with increasing BMI in both men and women (**Table 2**). The highest prevalence rates of MAFLD were observed in males with obesity (66.14%) and in females with T2DM (64.54%). Additionally, males with obesity, HTN, and dyslipidemia had higher MAFLD rates than the females (all *p*<0.0001). Conversely, the prevalence of MAFLD in males with T2DM was significantly lower than that in females (*p*<0.0001; **Table 3**).

**Table 2:** Prevalence of MAFLD in different BMI groups stratified by sex.

Group	BMI<23, %, (n)	23≤BMI<25, %, (n)	BMI≥25, %, (n)
	(n=25,268)	(n=17,257)	(n=33,045)
Total	4.02 (n=1,016)	32.03 (n=5,528)	65.55 (n=21,662)
Female	3.55 (n=554)	29.74 (n=1,846)	63.41 (n=4,525)
Male	4.79 (n=462)	33.32 (n=3,682)	66.14 (n=17,137)
P-value	<0.0001	<0.0001	<0.0001

P value for comparison between female and male.

BMI (kg/m_2_), body mass index.

**Table 3:** Prevalence of MAFLD in participants with different metabolic disorders stratified by sex.

Group	Obesity, %, (n)	T2DM, %, (n)	HTN, %, (n)	Dyslipidemia, %, (n)
	(n=33,045)	(n=4,847)	(n=22,008)	(n=38,916)
Total	65.55 (n=21662)	61.56 (n=2,984)	54.06 (n=11,898)	52.20 (n=20,313)
Female	63.41 (n=4,525)	64.54 (n=659)	47.82 (n=2,692)	39.65 (n=4,565)
Male	66.14 (n=17,137)	60.77 (n=2,325)	56.21 (n=9,206)	57.47 (n=15,748)
P value	<0.0001	0.028	<0.0001	<0.0001

P value for comparison between female and male.

T2DM, type 2 diabetes mellitus; HTN, hypertension.

### 3.3 Risk factors for MAFLD

**Figure 3:**
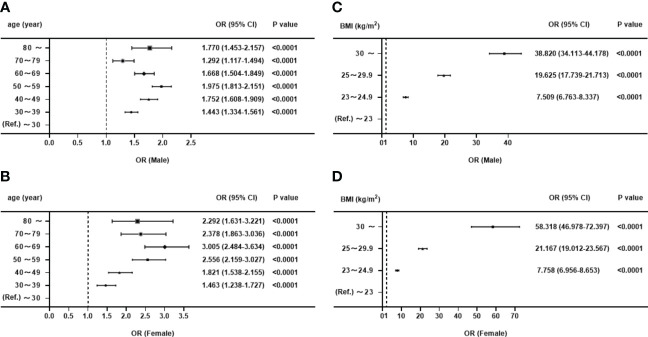
Forest map for binary logistic regression analysis of women and men. **(A)** OR for the association between age and MAFLD in men. **(B)** OR for the association between age and MAFLD in women. **(C)** OR for the association between BMI and MAFLD in men. **(D)** OR for the association between BMI and MAFLD in women.

In both males and females, DBP, FPG, TG, ALT, GGT, and UA were positively associated with the ORs of MALFD, whereas HDL-C was negatively associated with the ORs of MALFD (all *p*<0.0001). The relationship between age, BMI, and MAFLD is presented in **Figure 3**, which shows the incidence of MAFLD across various age groups and BMI categories. Independent of sex, each age group was associated with MAFLD incidence, with a higher risk observed in older individuals. Among women, the highest risk of MAFLD was observed in the 60–69 years age group (OR=3.005; 95% CI: 2.484–3.634), whereas for men, the risk tended to be similar across all age groups, except for those aged 50–59 years (OR=1.975, 95% CI: 1.813–2.151). Accounting for potential confounding factors, the risk of MAFLD increased with an increase in BMI. For individuals with BMI <25 kg/m2, the risk of MAFLD was slightly higher in women than in men. However, in those with BMI ≥25 kg/m2, the risk of MAFLD in men is significantly higher than that in women. Notably, the risk increased significantly for both men and women when the BMI was ≥30 kg/m^2^, and reached ORs of 38.82 (95% CI: 34.113–44.178) for men and 58.318 (95% CI: 46.978–72.397) for women.

## 4 Discussion

This cross-sectional study investigated the epidemiological characteristics of MAFLD and its associated risk factors in a large population in Xuzhou, China. Our findings revealed that MAFLD poses a significant burden on the Chinese population undergoing health check-ups, with an overall prevalence of 37.32%. Notably, we observed a sex-specific difference in MAFLD prevalence, with men having a higher prevalence (45.66%) than women (23.91%). This indicated that men were more susceptible to MAFLD. In addition, our subgroup analysis based on age, BMI, and metabolic disorder showed that these sex-specific differences still existed, with males being more vulnerable to MAFLD than females at any given BMI, age group before 65 years, and different metabolic states. Furthermore, our data revealed a close association between MAFLD and BMI, as well as age, with participants who had a higher BMI and age having a significantly higher prevalence. These findings suggest that increased BMI and age may play a significant role in the onset and development of MAFLD.

First and foremost, we would like to discuss the particular division of our study groups. According to the new definition, hepatic steatosis is a prerequisite for MAFLD. Consequently, patients who do not have hepatic steatosis or have hepatic steatosis without a metabolic disorder should be classified as non-MAFLD individuals. In theory, our study should perform a comparison between those general non-MAFLD individuals and MAFLD patients. However, our study primarily focuses on investigating the influence of metabolic disorders on the prevalence of hepatic steatosis in light of the new definition of MAFLD and comparing the epidemiological variances between hepatic steatosis patients with and without metabolic disorders. Therefore, instead of comparing the general non-MAFLD group, we chose to compare a specific group of non-MAFLD (hepatic steatosis without metabolic disorder) against the MAFLD group.

China has seen a significant increase in the prevalence of MAFLD over the past few decades – from 23.8% in 2001 to 32.9% in 2018 (15). This trend is expected to continue because of the increasing prevalence of obesity, diabetes, and metabolic syndrome. By 2030, it is projected that there will be approximately 314.58 million cases of MAFLD, indicating that the disease will have a profound impact in the years to come (6). Our study found a prevalence rate of 37.32%, which is consistent with the reported prevalence of MAFLD (38.1%) in the United States (16). A meta-analysis of observational data also found pooled prevalence rates of 39.22% and 33.86%, respectively (17). The new term “MAFLD” could potentially explain the increased occurrence of the disease in individuals who have both excessive alcohol consumption or viral infections in addition to metabolic disorders. Furthermore, our study revealed a clear male predominance in the prevalence of MAFLD, with a markedly higher estimated prevalence in males (45.66%) than in females (23.91%). This result is consistent with that of a recent nationwide study involving 2,083,984 participants (18). However, another study suggested a higher frequency of MAFLD in women (31.7%) than in men (25.5%), which may be due to a smaller sample size and different age groups in the study population (8). This study included only 5,377 participants with a mean age of 67 years, which may have included a high proportion of estrogen-deficient postmenopausal women, although estrogen has been shown to be a protective factor against MAFLD (19). Overall, our study and previous studies have consistently shown significant sex-specific differences in the prevalence of MAFLD.

Aging has been linked to various metabolic disorders in numerous studies (20). To examine the impact of age and sex on the prevalence of MAFLD, we categorized the participants based on age and sex. Our findings indicate that the prevalence of MAFLD is not linearly associated with age but is affected by sex. The highest prevalence of MAFLD was observed in the quinquagenarian (50-59 years old) group, suggesting that MAFLD is more prevalent in middle-aged individuals. Similarly, the prevalence of MAFLD in males peaks at approximately 50 years of age and declines thereafter. Possible reasons include the following: middle-aged men may face higher stress and social obligations that lead to unhealthy habits, such as excessive drinking, which increases their risk of developing metabolic diseases. In contrast, women showed a slow increase in susceptibility to MAFLD until the age of 50 years, after which it accelerated sharply. This sex difference in the peak prevalence suggests that a decrease in estrogen levels may be the primary cause of the sudden increase in MAFLD prevalence in older women. Low estrogen levels during the postmenopausal period may be an important risk factor for MAFLD in women, as several studies have shown that decreased estrogen levels are associated with metabolic disorders (21, 22). Moreover, decreased estrogen levels can cause an increase in pro-inflammatory cytokine levels and decrease hepatic insulin clearance, allowing the development of diet-induced insulin resistance, thus promoting the formation of MAFLD (23, 24). Finally, we recommend paying special attention to the young male generation (18-39 years old), as they have experienced the fastest growth rate in MAFLD prevalence. Early intervention effectively reduced the risk of MAFLD.

Our study found a positive correlation between BMI and prevalence of MAFLD, with men having a higher prevalence of MAFLD than women across all BMI categories. Obesity is a major independent and modifiable risk factor for MAFLD, which is supported by numerous epidemiological studies linking an increasing obesity rate to a simultaneous increase in the prevalence of MAFLD (25, 26). Chang et al. also discovered a strong approximately linear relationship between the incidence of fatty liver and an increased baseline BMI. Regardless of metabolic abnormalities, patients with obesity have a higher risk of developing fatty liver (27). Our study confirmed that overweight and obesity were the primary risk factors for MAFLD in both men and women. To better understand the association between the severity of obesity and MAFLD, we divided obesity into moderate and severe obesity categories. Compared to normal-weight individuals, we discovered that the prevalence of MAFLD was 38.82 and 58.318 times higher in male and female patients with severe obesity, respectively, indicating that women with severe obesity are at a higher risk of developing MAFLD. The reasons for the stronger association between BMI and incident MAFLD in female patients with obesity are unclear; however, excess adiposity may be a more significant contributor to fatty liver in women than in men. Further research is required to fully understand these sex-related differences. These findings highlight the close association between increased BMI and MAFLD and emphasize the importance of weight management. As outlined in the guidelines for MAFLD management, lifestyle interventions play a key role in the primary and secondary prevention of MAFLD (28). Most lifestyle interventions achieve a weight loss of 5% to 10%, leading to significant improvements in hepatic histological features (steatosis, inflammation, and fibrosis) and a reduction in cardiovascular disease risk (29, 30).

Our study revealed a high prevalence of metabolic abnormalities in patients with MAFLD. Furthermore, patients with metabolic disorders have a higher prevalence of MAFLD. These results suggest that MAFLD is closely associated with systemic metabolic disorders, indicating a multifaceted relationship between MAFLD and MetS. It is also noteworthy that MAFLD can better predict disease progression and severity than NAFLD (9). Another interesting finding from our study is that males with obesity, HTN, and dyslipidemia exhibited higher MAFLD rates than females. This suggests that males with these metabolic dysfunctions may be more vulnerable to MAFLD and indicates the need for further study. In contrast, females with T2DM had higher rates of MAFLD, likely because of the interaction between T2DM and MAFLD. Similarly, a previous study in Scotland based on hospital and death records spanning over 10 years found that males with T2DM were three times more likely to develop NAFLD, while females had a five-fold increased risk of NAFLD (31).

Nonetheless, the limitations of this study warrant further investigation. First, MAFLD diagnosis relies on ultrasonography, which may have reduced the sensitivity when liver steatosis is <30% (32). Therefore, using ultrasound to screen for MAFLD may have underestimated the true prevalence of MAFLD, although the possibly underestimated value in our study has already demonstrated the heavy burden of MAFLD in China. Second, we did not measure 2-h postload blood glucose, HbA1c, waist circumference, insulin resistance index, or high-sensitivity C-reactive protein levels, which are necessary components for defining metabolic disorders and MAFLD. Furthermore, accurate records of lifestyle factors or dietary habits were not well documented in this large retrospective database, which were also potential risk factors and could help us to better understand the natural history of the disease. Third, our study did not record the estrogen levels or menopausal status, which are essential factors for understanding the correlation between sex-specific differences in the prevalence of MAFLD. Lastly, selection bias was inherent, as we only included asymptomatic individuals from a single center. In conclusion, multicenter prospective studies with larger populations and more diverse potential risk factors, including lifestyle factors and dietary habits, are required to fully support and validate our findings.

## 5 Conclusion

Our findings suggest a high prevalence of MAFLD and a high prevalence of other metabolic disorders, particularly obesity, in the population. These results contribute to the understanding of the epidemiological characteristics of MAFLD based on health checkups in China. It is essential to focus on preventing and managing multiple metabolic disorders, particularly obesity, to effectively treat MAFLD. These findings have significant implications for the development of personalized interventions to prevent and treat MAFLD in high-risk groups.

## Data availability statement

The original contributions presented in the study are included in the article/supplementary materials. Further inquiries can be directed to the corresponding authors.

## Ethics statement

The studies involving humans were approved by the Ethics Committee of the Affiliated Hospital of Xuzhou Medical University. The studies were conducted in accordance with the local legislation and institutional requirements. The requirement for informed consent was waived because of deidentified data were analyzed in this retrospective study.

## Author contributions

MC, PS, and GS conceived and designed the study. MC, ZS, and WW coordinated data collection and conducted the analyses. MC wrote the manuscript. All authors contributed to the article and approved the submitted version.
